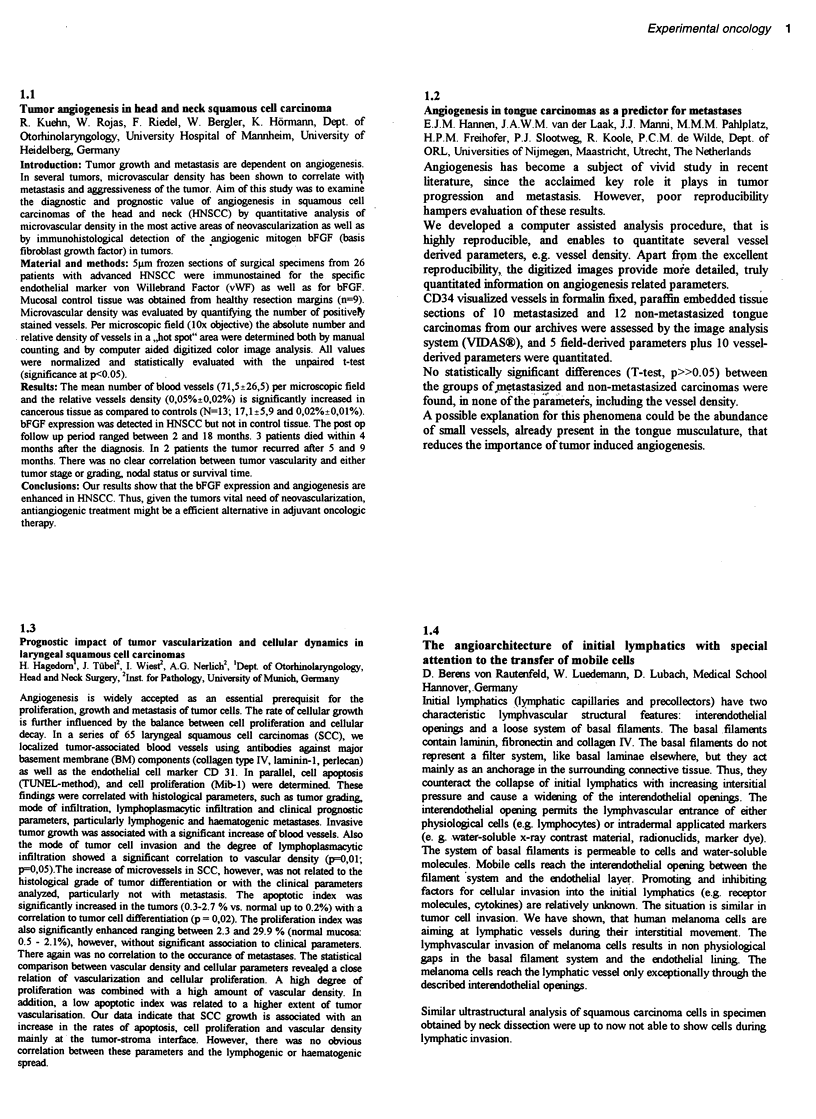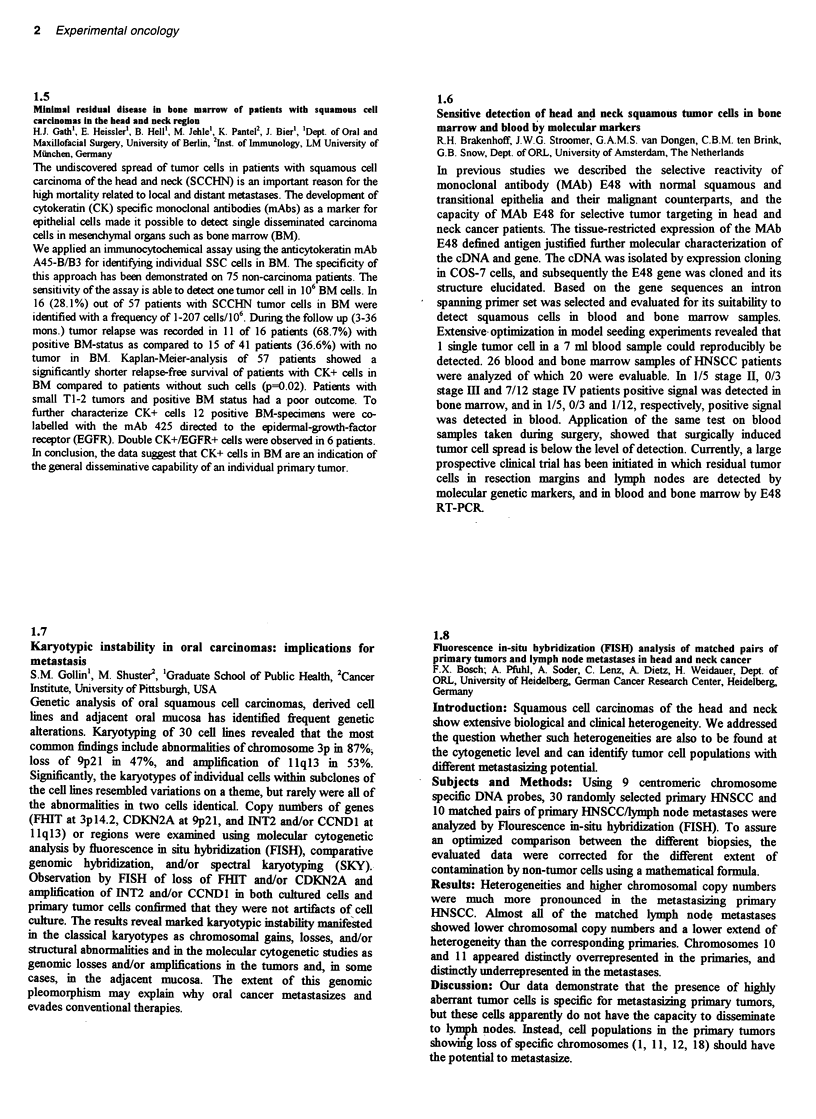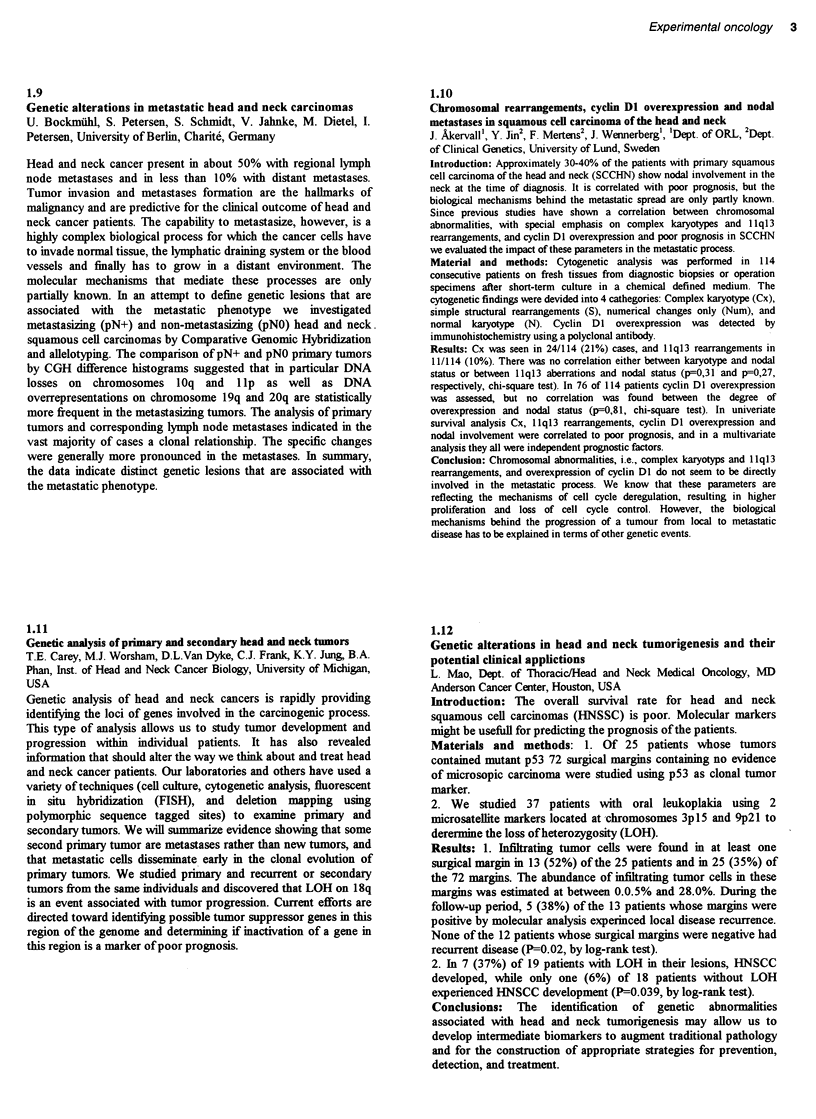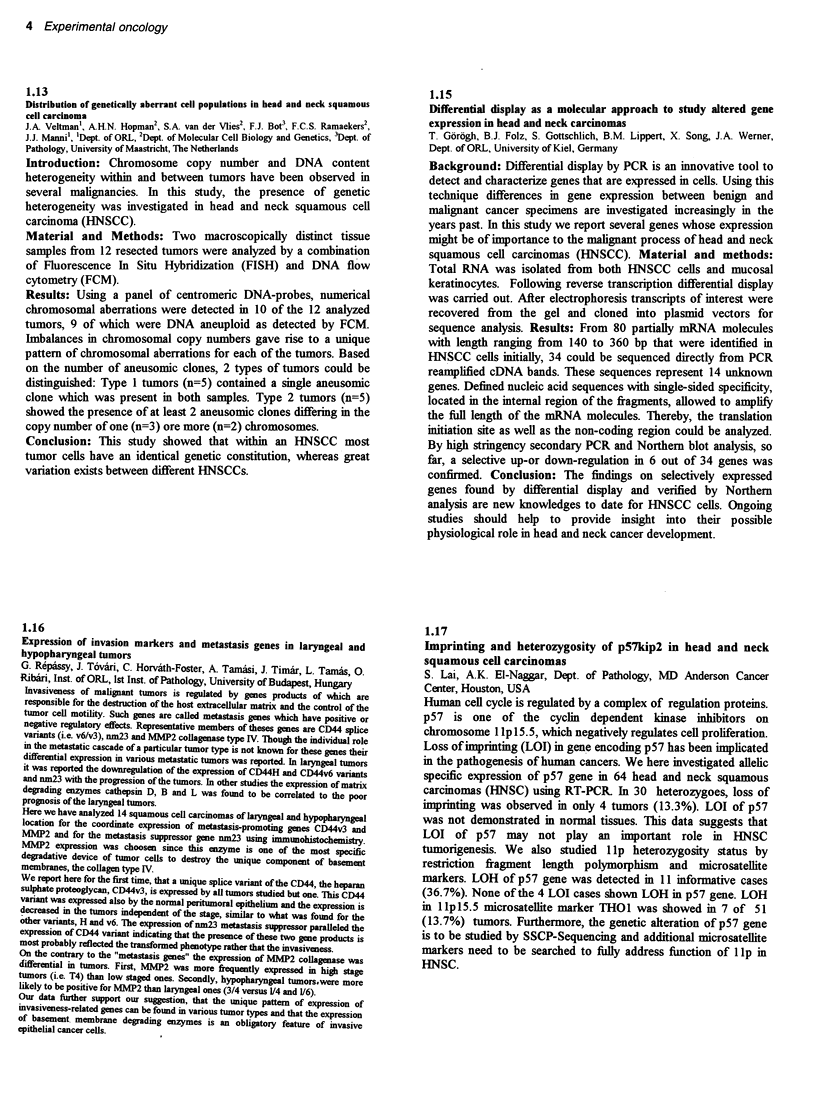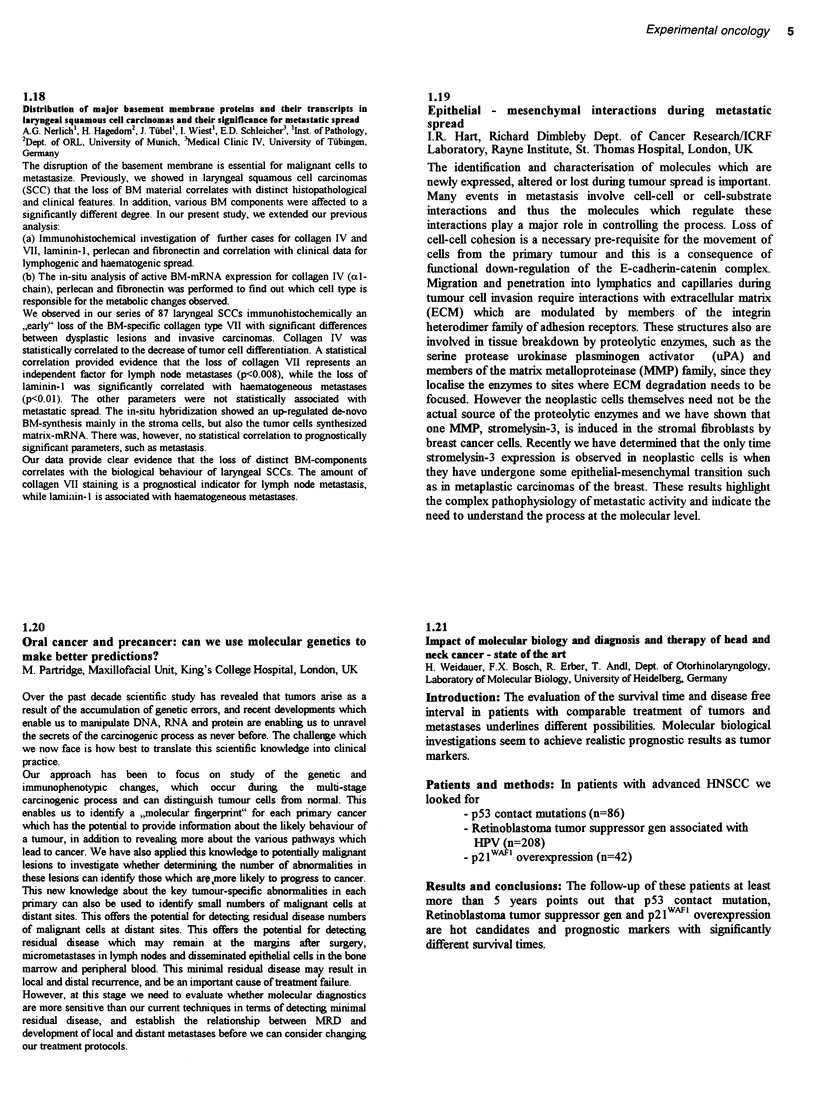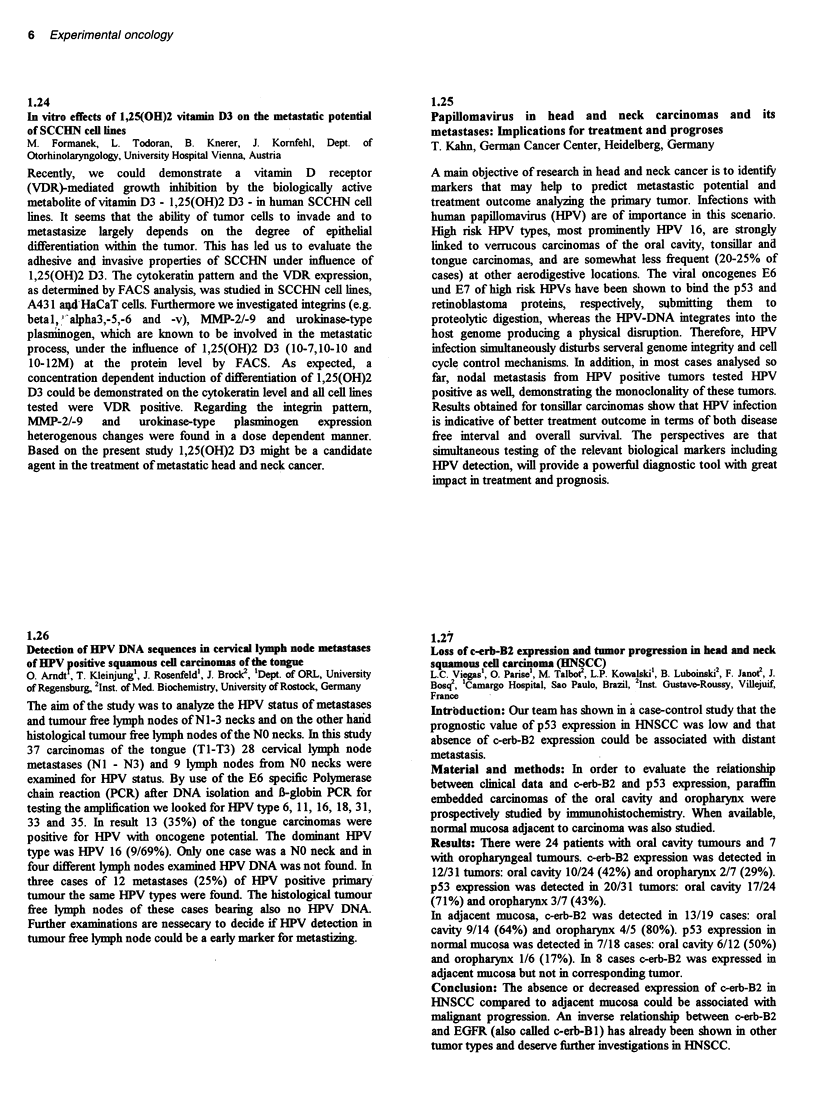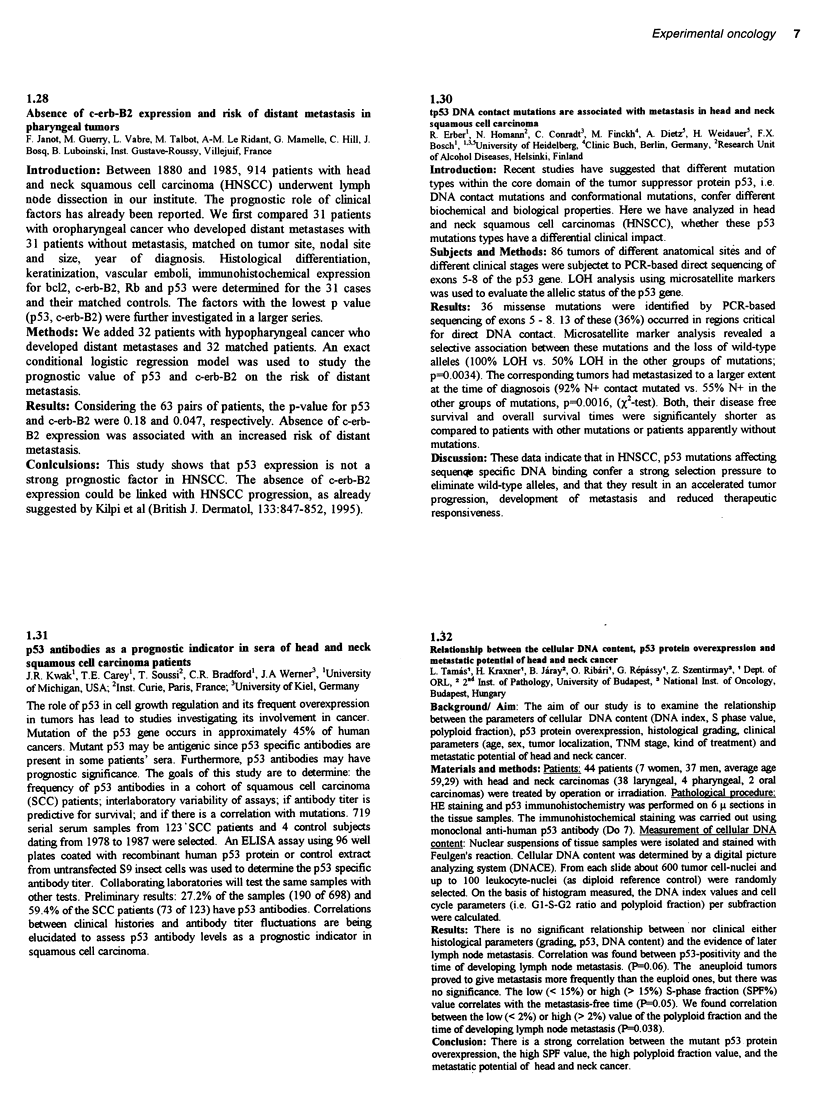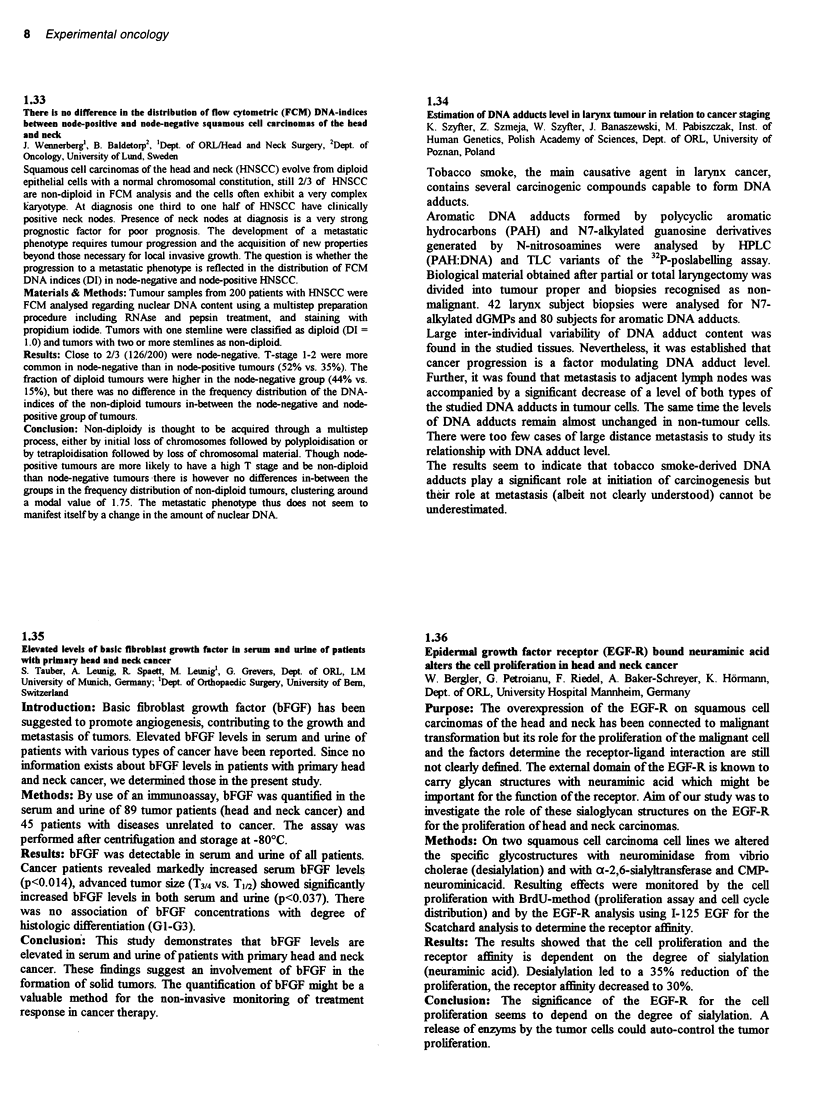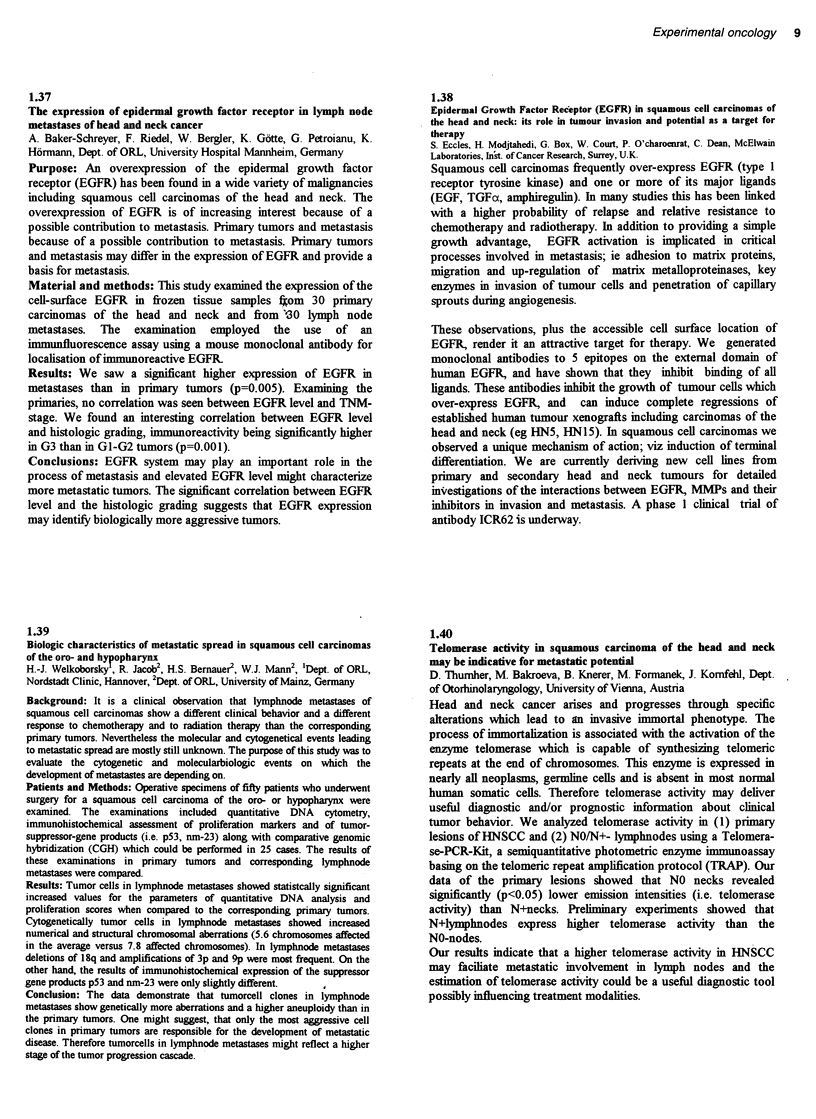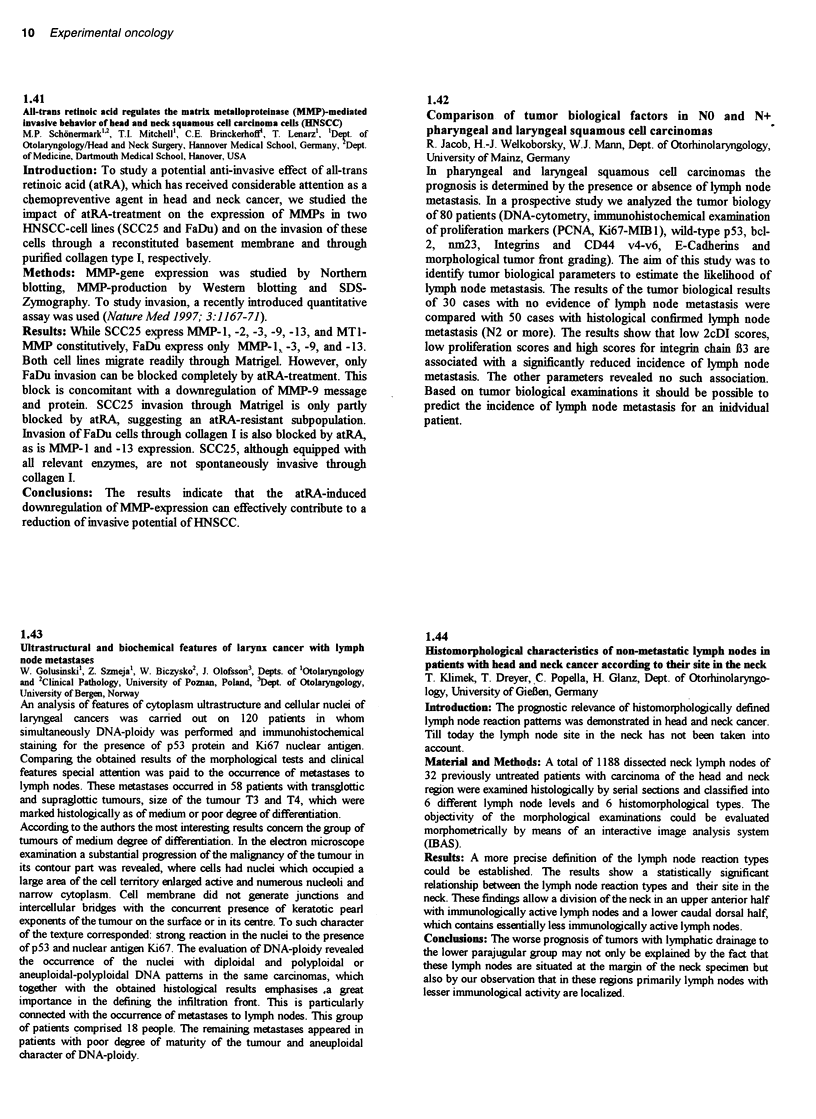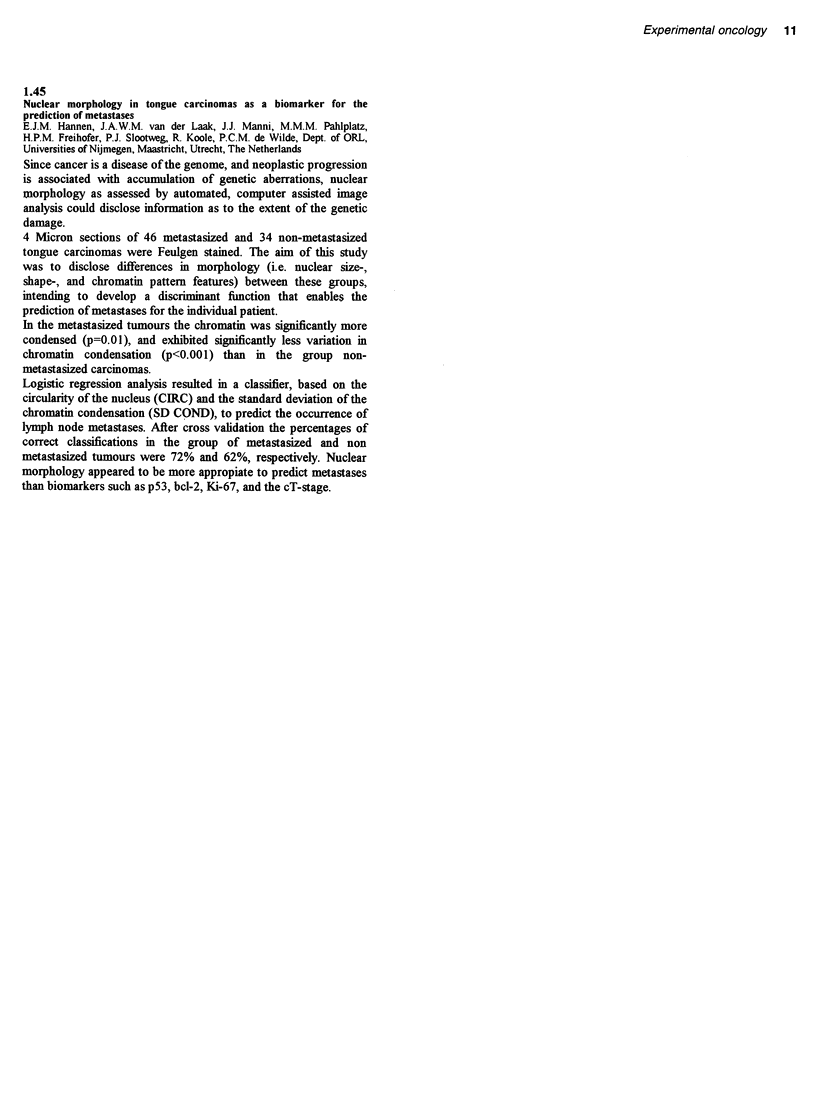# International Symposium on Metastases in Head and Neck Cancer. Kiel, Germany, 15-18 January 1998. Abstracts.

**Published:** 1998

**Authors:** 


### Tumor angiogenesis in head and neck squamous cell carcinoma

R. Kuehn, W. Rojas, F. Riedel, W. Bergler, K. Hörmann, Dept. of Otorhinolaryngology, University Hospital of Mannheim, University of Heidelberg, Germany

**Introduction:** Tumor growth and metastasis are dependent on angiogenesis. In several tumors, microvascular density has been shown to correlate with metastasis and aggressiveness of the tumor. Aim of this study was to examine the diagnostic and prognostic value of angiogenesis in squamous cell carcinomas of the head and neck (HNSCC) by quantitative analysis of microvascular density in the most active areas of neovascularization as well as by immunohistological detection of the angiogenic mitogen bFGF (basis fibroblast growth factor) in tumors.

**Material and methods:** 5µm frozen sections of surgical specimens from 26 patients with advanced HNSCC were immunostained for the specific endothelial marker von Willebrand Factor (vWF) as well as for bFGF. Mucosal control tissue was obtained from healthy resection margins (n=9). Microvascular density was evaluated by quantifying the number of positively stained vessels. Per microscopic field (10x objective) the absolute number and relative density of vessels in a „hot spot" area were determined both by manual counting and by computer aided digitized color image analysis. All values were normalized and statistically evaluated with the unpaired t-test (significance at $p<0.05$).

**Results:** The mean number of blood vessels (71,5±26,5) per microscopic field and the relative vessels density (0,05%±0,02%) is significantly increased in cancerous tissue as compared to controls (N=13; 17,1±5,9 and 0,02%±0,01%). bFGF expression was detected in HNSCC but not in control tissue. The post op follow up period ranged between 2 and 18 months. 3 patients died within 4 months after the diagnosis. In 2 patients the tumor recurred after 5 and 9 months. There was no clear correlation between tumor vascularity and either tumor stage or grading, nodal status or survival time.

**Conclusions:** Our results show that the bFGF expression and angiogenesis are enhanced in HNSCC. Thus, given the tumors vital need of neovascularization, antiangiogenic treatment might be a efficient alternative in adjuvant oncologic therapy.

### 1.2

### Angiogenesis in tongue carcinomas as a predictor for metastases

E.J.M. Hannen, J.A.W.M. van der Laak, J.J. Manni, M.M.M. Pahlplatz, H.P.M. Freihofer, P.J. Slootweg, R. Koole, P.C.M. de Wilde, Dept. of ORL, Universities of Nijmegen, Maastricht, Utrecht, The Netherlands

Angiogenesis has become a subject of vivid study in recent literature, since the acclaimed key role it plays in tumor progression and metastasis. However, poor reproducibility hampers evaluation of these results.

We developed a computer assisted analysis procedure, that is highly reproducible, and enables to quantitate several vessel derived parameters, e.g. vessel density. Apart from the excellent reproducibility, the digitized images provide more detailed, truly quantitated information on angiogenesis related parameters.

CD34 visualized vessels in formalin fixed, paraffin embedded tissue sections of 10 metastasized and 12 non-metastasized tongue carcinomas from our archives were assessed by the image analysis system (VIDAS®), and 5 field-derived parameters plus 10 vessel-derived parameters were quantitated.

No statistically significant differences (T-test, $p>>0.05$) between the groups of metastasized and non-metastasized carcinomas were found, in none of the parameters, including the vessel density.

A possible explanation for this phenomena could be the abundance of small vessels, already present in the tongue musculature, that reduces the importance of tumor induced angiogenesis.

### 1.3

### Prognostic impact of tumor vascularization and cellular dynamics in laryngeal squamous cell carcinomas

H. Hagedorn[1], J. Tübel[2], I. Wiest[2], A.G. Nerlich[2], [1]Dept. of Otorhinolaryngology, Head and Neck Surgery, [2]Inst. for Pathology, University of Munich, Germany

Angiogenesis is widely accepted as an essential prerequisit for the proliferation, growth and metastasis of tumor cells. The rate of cellular growth is further influenced by the balance between cell proliferation and cellular decay. In a series of 65 laryngeal squamous cell carcinomas (SCC), we localized tumor-associated blood vessels using antibodies against major basement membrane (BM) components (collagen type IV, laminin-1, perlecan) as well as the endothelial cell marker CD 31. In parallel, cell apoptosis (TUNEL-method), and cell proliferation (Mib-1) were determined. These findings were correlated with histological parameters, such as tumor grading, mode of infiltration, lymphoplasmacytic infiltration and clinical prognostic parameters, particularly lymphogenic and haematogenic metastases. Invasive tumor growth was associated with a significant increase of blood vessels. Also the mode of tumor cell invasion and the degree of lymphoplasmacytic infiltration showed a significant correlation to vascular density ($p=0,01$; $p=0,05$).The increase of microvessels in SCC, however, was not related to the histological grade of tumor differentiation or with the clinical parameters analyzed, particularly not with metastasis. The apoptotic index was significantly increased in the tumors (0.3-2.7 % vs. normal up to 0.2%) with a correlation to tumor cell differentiation ($p = 0,02$). The proliferation index was also significantly enhanced ranging between 2.3 and 29.9 % (normal mucosa: 0.5 - 2.1%), however, without significant association to clinical parameters. There again was no correlation to the occurance of metastases. The statistical comparison between vascular density and cellular parameters revealed a close relation of vascularization and cellular proliferation. A high degree of proliferation was combined with a high amount of vascular density. In addition, a low apoptotic index was related to a higher extent of tumor vascularisation. Our data indicate that SCC growth is associated with an increase in the rates of apoptosis, cell proliferation and vascular density mainly at the tumor-stroma interface. However, there was no obvious correlation between these parameters and the lymphogenic or haematogenic spread.

### 1.4

### The angioarchitecture of initial lymphatics with special attention to the transfer of mobile cells

D. Berens von Rautenfeld, W. Luedemann, D. Lubach, Medical School Hannover, Germany

Initial lymphatics (lymphatic capillaries and precollectors) have two characteristic lymphvascular structural features: interendothelial openings and a loose system of basal filaments. The basal filaments contain laminin, fibronectin and collagen IV. The basal filaments do not represent a filter system, like basal laminae elsewhere, but they act mainly as an anchorage in the surrounding connective tissue. Thus, they counteract the collapse of initial lymphatics with increasing intersitial pressure and cause a widening of the interendothelial openings. The interendothelial opening permits the lymphvascular entrance of either physiological cells (e.g. lymphocytes) or intradermal applicated markers (e. g. water-soluble x-ray contrast material, radionuclids, marker dye). The system of basal filaments is permeable to cells and water-soluble molecules. Mobile cells reach the interendothelial opening between the filament system and the endothelial layer. Promoting and inhibiting factors for cellular invasion into the initial lymphatics (e.g. receptor molecules, cytokines) are relatively unknown. The situation is similar in tumor cell invasion. We have shown, that human melanoma cells are aiming at lymphatic vessels during their interstitial movement. The lymphvascular invasion of melanoma cells results in non physiological gaps in the basal filament system and the endothelial lining. The melanoma cells reach the lymphatic vessel only exceptionally through the described interendothelial openings.

Similar ultrastructural analysis of squamous carcinoma cells in specimen obtained by neck dissection were up to now not able to show cells during lymphatic invasion.

### 1.5

### Minimal residual disease in bone marrow of patients with squamous cell carcinomas in the head and neck region

H.J. Gath[1], E. Heissler[1], B. Hell[1], M. Jehle[1], K. Pantel[2], J. Bier[1], [1]Dept. of Oral and Maxillofacial Surgery, University of Berlin, [2]Inst. of Immunology, LM University of München, Germany

The undiscovered spread of tumor cells in patients with squamous cell carcinoma of the head and neck (SCCHN) is an important reason for the high mortality related to local and distant metastases. The development of cytokeratin (CK) specific monoclonal antibodies (mAbs) as a marker for epithelial cells made it possible to detect single disseminated carcinoma cells in mesenchymal organs such as bone marrow (BM).

We applied an immunocytochemical assay using the anticytokeratin mAb A45-B/B3 for identifying individual SSC cells in BM. The specificity of this approach has been demonstrated on 75 non-carcinoma patients. The sensitivity of the assay is able to detect one tumor cell in $10^6$ BM cells. In 16 (28.1%) out of 57 patients with SCCHN tumor cells in BM were identified with a frequency of 1-207 cells/$10^6$. During the follow up (3-36 mons.) tumor relapse was recorded in 11 of 16 patients (68.7%) with positive BM-status as compared to 15 of 41 patients (36.6%) with no tumor in BM. Kaplan-Meier-analysis of 57 patients showed a significantly shorter relapse-free survival of patients with CK+ cells in BM compared to patients without such cells (p=0.02). Patients with small T1-2 tumors and positive BM status had a poor outcome. To further characterize CK+ cells 12 positive BM-specimens were co-labelled with the mAb 425 directed to the epidermal-growth-factor receptor (EGFR). Double CK+/EGFR+ cells were observed in 6 patients. In conclusion, the data suggest that CK+ cells in BM are an indication of the general disseminative capability of an individual primary tumor.

### 1.6

### Sensitive detection of head and neck squamous tumor cells in bone marrow and blood by molecular markers

R.H. Brakenhoff, J.W.G. Stroomer, G.A.M.S. van Dongen, C.B.M. ten Brink, G.B. Snow, Dept. of ORL, University of Amsterdam, The Netherlands

In previous studies we described the selective reactivity of monoclonal antibody (MAb) E48 with normal squamous and transitional epithelia and their malignant counterparts, and the capacity of MAb E48 for selective tumor targeting in head and neck cancer patients. The tissue-restricted expression of the MAb E48 defined antigen justified further molecular characterization of the cDNA and gene. The cDNA was isolated by expression cloning in COS-7 cells, and subsequently the E48 gene was cloned and its structure elucidated. Based on the gene sequences an intron spanning primer set was selected and evaluated for its suitability to detect squamous cells in blood and bone marrow samples. Extensive optimization in model seeding experiments revealed that 1 single tumor cell in a 7 ml blood sample could reproducibly be detected. 26 blood and bone marrow samples of HNSCC patients were analyzed of which 20 were evaluable. In 1/5 stage II, 0/3 stage III and 7/12 stage IV patients positive signal was detected in bone marrow, and in 1/5, 0/3 and 1/12, respectively, positive signal was detected in blood. Application of the same test on blood samples taken during surgery, showed that surgically induced tumor cell spread is below the level of detection. Currently, a large prospective clinical trial has been initiated in which residual tumor cells in resection margins and lymph nodes are detected by molecular genetic markers, and in blood and bone marrow by E48 RT-PCR.

### 1.7

### Karyotypic instability in oral carcinomas: implications for metastasis

S.M. Gollin[1], M. Shuster[2], [1]Graduate School of Public Health, [2]Cancer Institute, University of Pittsburgh, USA

Genetic analysis of oral squamous cell carcinomas, derived cell lines and adjacent oral mucosa has identified frequent genetic alterations. Karyotyping of 30 cell lines revealed that the most common findings include abnormalities of chromosome 3p in 87%, loss of 9p21 in 47%, and amplification of 11q13 in 53%. Significantly, the karyotypes of individual cells within subclones of the cell lines resembled variations on a theme, but rarely were all of the abnormalities in two cells identical. Copy numbers of genes (FHIT at 3p14.2, CDKN2A at 9p21, and INT2 and/or CCND1 at 11q13) or regions were examined using molecular cytogenetic analysis by fluorescence in situ hybridization (FISH), comparative genomic hybridization, and/or spectral karyotyping (SKY). Observation by FISH of loss of FHIT and/or CDKN2A and amplification of INT2 and/or CCND1 in both cultured cells and primary tumor cells confirmed that they were not artifacts of cell culture. The results reveal marked karyotypic instability manifested in the classical karyotypes as chromosomal gains, losses, and/or structural abnormalities and in the molecular cytogenetic studies as genomic losses and/or amplifications in the tumors and, in some cases, in the adjacent mucosa. The extent of this genomic pleomorphism may explain why oral cancer metastasizes and evades conventional therapies.

### 1.8

### Fluorescence in-situ hybridization (FISH) analysis of matched pairs of primary tumors and lymph node metastases in head and neck cancer

F.X. Bosch; A. Pfuhl, A. Soder, C. Lenz, A. Dietz, H. Weidauer, Dept. of ORL, University of Heidelberg, German Cancer Research Center, Heidelberg, Germany

**Introduction:** Squamous cell carcinomas of the head and neck show extensive biological and clinical heterogeneity. We addressed the question whether such heterogeneities are also to be found at the cytogenetic level and can identify tumor cell populations with different metastasizing potential.

**Subjects and Methods:** Using 9 centromeric chromosome specific DNA probes, 30 randomly selected primary HNSCC and 10 matched pairs of primary HNSCC/lymph node metastases were analyzed by Flourescence in-situ hybridization (FISH). To assure an optimized comparison between the different biopsies, the evaluated data were corrected for the different extent of contamination by non-tumor cells using a mathematical formula.

**Results:** Heterogeneities and higher chromosomal copy numbers were much more pronounced in the metastasizing primary HNSCC. Almost all of the matched lymph node metastases showed lower chromosomal copy numbers and a lower extend of heterogeneity than the corresponding primaries. Chromosomes 10 and 11 appeared distinctly overrepresented in the primaries, and distinctly underrepresented in the metastases.

**Discussion:** Our data demonstrate that the presence of highly aberrant tumor cells is specific for metastasizing primary tumors, but these cells apparently do not have the capacity to disseminate to lymph nodes. Instead, cell populations in the primary tumors showing loss of specific chromosomes (1, 11, 12, 18) should have the potential to metastasize.

### 1.9

**Genetic alterations in metastatic head and neck carcinomas**

U. Bockmühl, S. Petersen, S. Schmidt, V. Jahnke, M. Dietel, I. Petersen, University of Berlin, Charité, Germany

Head and neck cancer present in about 50% with regional lymph node metastases and in less than 10% with distant metastases. Tumor invasion and metastases formation are the hallmarks of malignancy and are predictive for the clinical outcome of head and neck cancer patients. The capability to metastasize, however, is a highly complex biological process for which the cancer cells have to invade normal tissue, the lymphatic draining system or the blood vessels and finally has to grow in a distant environment. The molecular mechanisms that mediate these processes are only partially known. In an attempt to define genetic lesions that are associated with the metastatic phenotype we investigated metastasizing (pN+) and non-metastasizing (pN0) head and neck squamous cell carcinomas by Comparative Genomic Hybridization and allelotyping. The comparison of pN+ and pN0 primary tumors by CGH difference histograms suggested that in particular DNA losses on chromosomes 10q and 11p as well as DNA overrepresentations on chromosome 19q and 20q are statistically more frequent in the metastasizing tumors. The analysis of primary tumors and corresponding lymph node metastases indicated in the vast majority of cases a clonal relationship. The specific changes were generally more pronounced in the metastases. In summary, the data indicate distinct genetic lesions that are associated with the metastatic phenotype.

### 1.10

**Chromosomal rearrangements, cyclin D1 overexpression and nodal metastases in squamous cell carcinoma of the head and neck**

J. Åkervall[1], Y. Jin[2], F. Mertens[2], J. Wennerberg[1], [1]Dept. of ORL, [2]Dept. of Clinical Genetics, University of Lund, Sweden

**Introduction:** Approximately 30-40% of the patients with primary squamous cell carcinoma of the head and neck (SCCHN) show nodal involvement in the neck at the time of diagnosis. It is correlated with poor prognosis, but the biological mechanisms behind the metastatic spread are only partly known. Since previous studies have shown a correlation between chromosomal abnormalities, with special emphasis on complex karyotypes and 11q13 rearrangements, and cyclin D1 overexpression and poor prognosis in SCCHN we evaluated the impact of these parameters in the metastatic process.

**Material and methods:** Cytogenetic analysis was performed in 114 consecutive patients on fresh tissues from diagnostic biopsies or operation specimens after short-term culture in a chemical defined medium. The cytogenetic findings were devided into 4 cathegories: Complex karyotype (Cx), simple structural rearrangements (S), numerical changes only (Num), and normal karyotype (N). Cyclin D1 overexpression was detected by immunohistochemistry using a polyclonal antibody.

**Results:** Cx was seen in 24/114 (21%) cases, and 11q13 rearrangements in 11/114 (10%). There was no correlation either between karyotype and nodal status or between 11q13 aberrations and nodal status (p=0,31 and p=0,27, respectively, chi-square test). In 76 of 114 patients cyclin D1 overexpression was assessed, but no correlation was found between the degree of overexpression and nodal status (p=0,81, chi-square test). In univeriate survival analysis Cx, 11q13 rearrangements, cyclin D1 overexpression and nodal involvement were correlated to poor prognosis, and in a multivariate analysis they all were independent prognostic factors.

**Conclusion:** Chromosomal abnormalities, i.e., complex karyotyps and 11q13 rearrangements, and overexpression of cyclin D1 do not seem to be directly involved in the metastatic process. We know that these parameters are reflecting the mechanisms of cell cycle deregulation, resulting in higher proliferation and loss of cell cycle control. However, the biological mechanisms behind the progression of a tumour from local to metastatic disease has to be explained in terms of other genetic events.

### 1.11

**Genetic analysis of primary and secondary head and neck tumors**

T.E. Carey, M.J. Worsham, D.L.Van Dyke, C.J. Frank, K.Y. Jung, B.A. Phan, Inst. of Head and Neck Cancer Biology, University of Michigan, USA

Genetic analysis of head and neck cancers is rapidly providing identifying the loci of genes involved in the carcinogenic process. This type of analysis allows us to study tumor development and progression within individual patients. It has also revealed information that should alter the way we think about and treat head and neck cancer patients. Our laboratories and others have used a variety of techniques (cell culture, cytogenetic analysis, fluorescent in situ hybridization (FISH), and deletion mapping using polymorphic sequence tagged sites) to examine primary and secondary tumors. We will summarize evidence showing that some second primary tumor are metastases rather than new tumors, and that metastatic cells disseminate early in the clonal evolution of primary tumors. We studied primary and recurrent or secondary tumors from the same individuals and discovered that LOH on 18q is an event associated with tumor progression. Current efforts are directed toward identifying possible tumor suppressor genes in this region of the genome and determining if inactivation of a gene in this region is a marker of poor prognosis.

### 1.12

**Genetic alterations in head and neck tumorigenesis and their potential clinical applictions**

L. Mao, Dept. of Thoracic/Head and Neck Medical Oncology, MD Anderson Cancer Center, Houston, USA

**Introduction:** The overall survival rate for head and neck squamous cell carcinomas (HNSSC) is poor. Molecular markers might be usefull for predicting the prognosis of the patients.

**Materials and methods**: 1. Of 25 patients whose tumors contained mutant p53 72 surgical margins containing no evidence of microsopic carcinoma were studied using p53 as clonal tumor marker.

2. We studied 37 patients with oral leukoplakia using 2 microsatellite markers located at chromosomes 3p15 and 9p21 to derermine the loss of heterozygosity (LOH).

**Results:** 1. Infiltrating tumor cells were found in at least one surgical margin in 13 (52%) of the 25 patients and in 25 (35%) of the 72 margins. The abundance of infiltrating tumor cells in these margins was estimated at between 0.0.5% and 28.0%. During the follow-up period, 5 (38%) of the 13 patients whose margins were positive by molecular analysis experinced local disease recurrence. None of the 12 patients whose surgical margins were negative had recurrent disease (P=0.02, by log-rank test).

2. In 7 (37%) of 19 patients with LOH in their lesions, HNSCC developed, while only one (6%) of 18 patients without LOH experienced HNSCC development (P=0.039, by log-rank test).

**Conclusions:** The identification of genetic abnormalities associated with head and neck tumorigenesis may allow us to develop intermediate biomarkers to augment traditional pathology and for the construction of appropriate strategies for prevention, detection, and treatment.

### 1.13

**Distribution of genetically aberrant cell populations in head and neck squamous cell carcinoma**

J.A. Veltman[1], A.H.N. Hopman[2], S.A. van der Vlies[2], F.J. Bot[3], F.C.S. Ramaekers[2], J.J. Manni[1], [1]Dept. of ORL, [2]Dept. of Molecular Cell Biology and Genetics, [3]Dept. of Pathology, University of Maastricht, The Netherlands

**Introduction:** Chromosome copy number and DNA content heterogeneity within and between tumors have been observed in several malignancies. In this study, the presence of genetic heterogeneity was investigated in head and neck squamous cell carcinoma (HNSCC).

**Material and Methods:** Two macroscopically distinct tissue samples from 12 resected tumors were analyzed by a combination of Fluorescence In Situ Hybridization (FISH) and DNA flow cytometry (FCM).

**Results:** Using a panel of centromeric DNA-probes, numerical chromosomal aberrations were detected in 10 of the 12 analyzed tumors, 9 of which were DNA aneuploid as detected by FCM. Imbalances in chromosomal copy numbers gave rise to a unique pattern of chromosomal aberrations for each of the tumors. Based on the number of aneusomic clones, 2 types of tumors could be distinguished: Type 1 tumors (n=5) contained a single aneusomic clone which was present in both samples. Type 2 tumors (n=5) showed the presence of at least 2 aneusomic clones differing in the copy number of one (n=3) ore more (n=2) chromosomes.

**Conclusion:** This study showed that within an HNSCC most tumor cells have an identical genetic constitution, whereas great variation exists between different HNSCCs.

### 1.15

**Differential display as a molecular approach to study altered gene expression in head and neck carcinomas**

T. Görögh, B.J. Folz, S. Gottschlich, B.M. Lippert, X. Song, J.A. Werner, Dept. of ORL, University of Kiel, Germany

**Background:** Differential display by PCR is an innovative tool to detect and characterize genes that are expressed in cells. Using this technique differences in gene expression between benign and malignant cancer specimens are investigated increasingly in the years past. In this study we report several genes whose expression might be of importance to the malignant process of head and neck squamous cell carcinomas (HNSCC). **Material and methods:** Total RNA was isolated from both HNSCC cells and mucosal keratinocytes. Following reverse transcription differential display was carried out. After electrophoresis transcripts of interest were recovered from the gel and cloned into plasmid vectors for sequence analysis. **Results:** From 80 partially mRNA molecules with length ranging from 140 to 360 bp that were identified in HNSCC cells initially, 34 could be sequenced directly from PCR reamplified cDNA bands. These sequences represent 14 unknown genes. Defined nucleic acid sequences with single-sided specificity, located in the internal region of the fragments, allowed to amplify the full length of the mRNA molecules. Thereby, the translation initiation site as well as the non-coding region could be analyzed. By high stringency secondary PCR and Northern blot analysis, so far, a selective up-or down-regulation in 6 out of 34 genes was confirmed. **Conclusion:** The findings on selectively expressed genes found by differential display and verified by Northern analysis are new knowledges to date for HNSCC cells. Ongoing studies should help to provide insight into their possible physiological role in head and neck cancer development.

### 1.16

**Expression of invasion markers and metastasis genes in laryngeal and hypopharyngeal tumors**

G. Répássy, J. Tóvári, C. Horváth-Foster, A. Tamási, J. Timár, L. Tamás, O. Ribári, Inst. of ORL, lst Inst. of Pathology, University of Budapest, Hungary

Invasiveness of malignant tumors is regulated by genes products of which are responsible for the destruction of the host extracellular matrix and the control of the tumor cell motility. Such genes are called metastasis genes which have positive or negative regulatory effects. Representative members of theses genes are CD44 splice variants (i.e. v6/v3), nm23 and MMP2 collagenase type IV. Though the individual role in the metastatic cascade of a particular tumor type is not known for these genes their differential expression in various metastatic tumors was reported. In laryngeal tumors it was reported the downregulation of the expression of CD44H and CD44v6 variants and nm23 with the progression of the tumors. In other studies the expression of matrix degrading enzymes cathepsin D, B and L was found to be correlated to the poor prognosis of the laryngeal tumors.

Here we have analyzed 14 squamous cell carcinomas of laryngeal and hypopharyngeal location for the coordinate expression of metastasis-promoting genes CD44v3 and MMP2 and for the metastasis suppressor gene nm23 using immunohistochemistry. MMP2 expression was choosen since this enzyme is one of the most specific degradative device of tumor cells to destroy the unique component of basement membranes, the collagen type IV.

We report here for the first time, that a unique splice variant of the CD44, the heparan sulphate proteoglycan, CD44v3, is expressed by all tumors studied but one. This CD44 variant was expressed also by the normal peritumoral epithelium and the expression is decreased in the tumors independent of the stage, similar to what was found for the other variants, H and v6. The expression of nm23 metastasis suppressor paralleled the expression of CD44 variant indicating that the presence of these two gene products is most probably reflected the transformed phenotype rather that the invasiveness.

On the contrary to the "metastasis genes" the expression of MMP2 collagenase was differential in tumors. First, MMP2 was more frequently expressed in high stage tumors (i.e. T4) than low staged ones. Secondly, hypopharyngeal tumors were more likely to be positive for MMP2 than laryngeal ones (3/4 versus l/4 and l/6).

Our data further support our suggestion, that the unique pattern of expression of invasiveness-related genes can be found in various tumor types and that the expression of basement membrane degrading enzymes is an obligatory feature of invasive epithelial cancer cells.

### 1.17

**Imprinting and heterozygosity of p57kip2 in head and neck squamous cell carcinomas**

S. Lai, A.K. El-Naggar, Dept. of Pathology, MD Anderson Cancer Center, Houston, USA

Human cell cycle is regulated by a complex of regulation proteins. p57 is one of the cyclin dependent kinase inhibitors on chromosome 11p15.5, which negatively regulates cell proliferation. Loss of imprinting (LOI) in gene encoding p57 has been implicated in the pathogenesis of human cancers. We here investigated allelic specific expression of p57 gene in 64 head and neck squamous carcinomas (HNSC) using RT-PCR. In 30 heterozygoes, loss of imprinting was observed in only 4 tumors (13.3%). LOI of p57 was not demonstrated in normal tissues. This data suggests that LOI of p57 may not play an important role in HNSC tumorigenesis. We also studied 11p heterozygosity status by restriction fragment length polymorphism and microsatellite markers. LOH of p57 gene was detected in 11 informative cases (36.7%). None of the 4 LOI cases shown LOH in p57 gene. LOH in 11p15.5 microsatellite marker THO1 was showed in 7 of 51 (13.7%) tumors. Furthermore, the genetic alteration of p57 gene is to be studied by SSCP-Sequencing and additional microsatellite markers need to be searched to fully address function of 11p in HNSC.

### 1.18

**Distribution of major basement membrane proteins and their transcripts in laryngeal squamous cell carcinomas and their significance for metastatic spread**

A.G. Nerlich[1], H. Hagedorn[2], J. Tübel[1], I. Wiest[1], E.D. Schleicher[3], [1]Inst. of Pathology, [2]Dept. of ORL, University of Munich, [3]Medical Clinic IV, University of Tübingen, Germany

The disruption of the basement membrane is essential for malignant cells to metastasize. Previously, we showed in laryngeal squamous cell carcinomas (SCC) that the loss of BM material correlates with distinct histopathological and clinical features. In addition, various BM components were affected to a significantly different degree. In our present study, we extended our previous analysis:

(a) Immunohistochemical investigation of further cases for collagen IV and VII, laminin-1, perlecan and fibronectin and correlation with clinical data for lymphogenic and haematogenic spread.

(b) The in-situ analysis of active BM-mRNA expression for collagen IV (α1-chain), perlecan and fibronectin was performed to find out which cell type is responsible for the metabolic changes observed.

We observed in our series of 87 laryngeal SCCs immunohistochemically an „early" loss of the BM-specific collagen type VII with significant differences between dysplastic lesions and invasive carcinomas. Collagen IV was statistically correlated to the decrease of tumor cell differentiation. A statistical correlation provided evidence that the loss of collagen VII represents an independent factor for lymph node metastases ($p<0.008$), while the loss of laminin-1 was significantly correlated with haematogeneous metastases ($p<0.01$). The other parameters were not statistically associated with metastatic spread. The in-situ hybridization showed an up-regulated de-novo BM-synthesis mainly in the stroma cells, but also the tumor cells synthesized matrix-mRNA. There was, however, no statistical correlation to prognostically significant parameters, such as metastasis.

Our data provide clear evidence that the loss of distinct BM-components correlates with the biological behaviour of laryngeal SCCs. The amount of collagen VII staining is a prognostical indicator for lymph node metastasis, while laminin-1 is associated with haematogeneous metastases.

### 1.19

**Epithelial - mesenchymal interactions during metastatic spread**

I.R. Hart, Richard Dimbleby Dept. of Cancer Research/ICRF Laboratory, Rayne Institute, St. Thomas Hospital, London, UK

The identification and characterisation of molecules which are newly expressed, altered or lost during tumour spread is important. Many events in metastasis involve cell-cell or cell-substrate interactions and thus the molecules which regulate these interactions play a major role in controlling the process. Loss of cell-cell cohesion is a necessary pre-requisite for the movement of cells from the primary tumour and this is a consequence of functional down-regulation of the E-cadherin-catenin complex. Migration and penetration into lymphatics and capillaries during tumour cell invasion require interactions with extracellular matrix (ECM) which are modulated by members of the integrin heterodimer family of adhesion receptors. These structures also are involved in tissue breakdown by proteolytic enzymes, such as the serine protease urokinase plasminogen activator (uPA) and members of the matrix metalloproteinase (MMP) family, since they localise the enzymes to sites where ECM degradation needs to be focused. However the neoplastic cells themselves need not be the actual source of the proteolytic enzymes and we have shown that one MMP, stromelysin-3, is induced in the stromal fibroblasts by breast cancer cells. Recently we have determined that the only time stromelysin-3 expression is observed in neoplastic cells is when they have undergone some epithelial-mesenchymal transition such as in metaplastic carcinomas of the breast. These results highlight the complex pathophysiology of metastatic activity and indicate the need to understand the process at the molecular level.

### 1.20

**Oral cancer and precancer: can we use molecular genetics to make better predictions?**

M. Partridge, Maxillofacial Unit, King's College Hospital, London, UK

Over the past decade scientific study has revealed that tumors arise as a result of the accumulation of genetic errors, and recent developments which enable us to manipulate DNA, RNA and protein are enabling us to unravel the secrets of the carcinogenic process as never before. The challenge which we now face is how best to translate this scientific knowledge into clinical practice.

Our approach has been to focus on study of the genetic and immunophenotypic changes, which occur during the multi-stage carcinogenic process and can distinguish tumour cells from normal. This enables us to identify a „molecular fingerprint" for each primary cancer which has the potential to provide information about the likely behaviour of a tumour, in addition to revealing more about the various pathways which lead to cancer. We have also applied this knowledge to potentially malignant lesions to investigate whether determining the number of abnormalities in these lesions can identify those which are more likely to progress to cancer. This new knowledge about the key tumour-specific abnormalities in each primary can also be used to identify small numbers of malignant cells at distant sites. This offers the potential for detecting residual disease numbers of malignant cells at distant sites. This offers the potential for detecting residual disease which may remain at the margins after surgery, micrometastases in lymph nodes and disseminated epithelial cells in the bone marrow and peripheral blood. This minimal residual disease may result in local and distal recurrence, and be an important cause of treatment failure.

However, at this stage we need to evaluate whether molecular diagnostics are more sensitive than our current techniques in terms of detecting minimal residual disease, and establish the relationship between MRD and development of local and distant metastases before we can consider changing our treatment protocols.

### 1.21

**Impact of molecular biology and diagnosis and therapy of head and neck cancer - state of the art**

H. Weidauer, F.X. Bosch, R. Erber, T. Andl, Dept. of Otorhinolaryngology, Laboratory of Molecular Biology, University of Heidelberg, Germany

**Introduction:** The evaluation of the survival time and disease free interval in patients with comparable treatment of tumors and metastases underlines different possibilities. Molecular biological investigations seem to achieve realistic prognostic results as tumor markers.

**Patients and methods:** In patients with advanced HNSCC we looked for

- p53 contact mutations (n=86)
- Retinoblastoma tumor suppressor gen associated with HPV (n=208)
- $p21^{WAF1}$ overexpression (n=42)

**Results and conclusions:** The follow-up of these patients at least more than 5 years points out that p53 contact mutation, Retinoblastoma tumor suppressor gen and $p21^{WAF1}$ overexpression are hot candidates and prognostic markers with significantly different survival times.

### 1.24

**In vitro effects of 1,25(OH)2 vitamin D3 on the metastatic potential of SCCHN cell lines**

M. Formanek, L. Todoran, B. Knerer, J. Kornfehl, Dept. of Otorhinolaryngology, University Hospital Vienna, Austria

Recently, we could demonstrate a vitamin D receptor (VDR)-mediated growth inhibition by the biologically active metabolite of vitamin D3 - 1,25(OH)2 D3 - in human SCCHN cell lines. It seems that the ability of tumor cells to invade and to metastasize largely depends on the degree of epithelial differentiation within the tumor. This has led us to evaluate the adhesive and invasive properties of SCCHN under influence of 1,25(OH)2 D3. The cytokeratin pattern and the VDR expression, as determined by FACS analysis, was studied in SCCHN cell lines, A431 and HaCaT cells. Furthermore we investigated integrins (e.g. beta1, alpha3,-5,-6 and -v), MMP-2/-9 and urokinase-type plasminogen, which are known to be involved in the metastatic process, under the influence of 1,25(OH)2 D3 (10-7,10-10 and 10-12M) at the protein level by FACS. As expected, a concentration dependent induction of differentiation of 1,25(OH)2 D3 could be demonstrated on the cytokeratin level and all cell lines tested were VDR positive. Regarding the integrin pattern, MMP-2/-9 and urokinase-type plasminogen expression heterogenous changes were found in a dose dependent manner. Based on the present study 1,25(OH)2 D3 might be a candidate agent in the treatment of metastatic head and neck cancer.

### 1.25

**Papillomavirus in head and neck carcinomas and its metastases: Implications for treatment and progroses**

T. Kahn, German Cancer Center, Heidelberg, Germany

A main objective of research in head and neck cancer is to identify markers that may help to predict metastastic potential and treatment outcome analyzing the primary tumor. Infections with human papillomavirus (HPV) are of importance in this scenario. High risk HPV types, most prominently HPV 16, are strongly linked to verrucous carcinomas of the oral cavity, tonsillar and tongue carcinomas, and are somewhat less frequent (20-25% of cases) at other aerodigestive locations. The viral oncogenes E6 und E7 of high risk HPVs have been shown to bind the p53 and retinoblastoma proteins, respectively, submitting them to proteolytic digestion, whereas the HPV-DNA integrates into the host genome producing a physical disruption. Therefore, HPV infection simultaneously disturbs serveral genome integrity and cell cycle control mechanisms. In addition, in most cases analysed so far, nodal metastasis from HPV positive tumors tested HPV positive as well, demonstrating the monoclonality of these tumors. Results obtained for tonsillar carcinomas show that HPV infection is indicative of better treatment outcome in terms of both disease free interval and overall survival. The perspectives are that simultaneous testing of the relevant biological markers including HPV detection, will provide a powerful diagnostic tool with great impact in treatment and prognosis.

### 1.26

**Detection of HPV DNA sequences in cervical lymph node metastases of HPV positive squamous cell carcinomas of the tongue**

O. Arndt[1], T. Kleinjung[1], J. Rosenfeld[1], J. Brock[2], [1]Dept. of ORL, University of Regensburg, [2]Inst. of Med. Biochemistry, University of Rostock, Germany

The aim of the study was to analyze the HPV status of metastases and tumour free lymph nodes of N1-3 necks and on the other hand histological tumour free lymph nodes of the N0 necks. In this study 37 carcinomas of the tongue (T1-T3) 28 cervical lymph node metastases (N1 - N3) and 9 lymph nodes from N0 necks were examined for HPV status. By use of the E6 specific Polymerase chain reaction (PCR) after DNA isolation and ß-globin PCR for testing the amplification we looked for HPV type 6, 11, 16, 18, 31, 33 and 35. In result 13 (35%) of the tongue carcinomas were positive for HPV with oncogene potential. The dominant HPV type was HPV 16 (9/69%). Only one case was a N0 neck and in four different lymph nodes examined HPV DNA was not found. In three cases of 12 metastases (25%) of HPV positive primary tumour the same HPV types were found. The histological tumour free lymph nodes of these cases bearing also no HPV DNA. Further examinations are nessecary to decide if HPV detection in tumour free lymph node could be a early marker for metastizing.

### 1.27

**Loss of c-erb-B2 expression and tumor progression in head and neck squamous cell carcinoma (HNSCC)**

L.C. Viegas[1], O. Parise[1], M. Talbot[2], L.P. Kowalski[1], B. Luboinski[2], F. Janot[2], J. Bosq[2], [1]Camargo Hospital, Sao Paulo, Brazil, [2]Inst. Gustave-Roussy, Villejuif, France

**Introduction:** Our team has shown in a case-control study that the prognostic value of p53 expression in HNSCC was low and that absence of c-erb-B2 expression could be associated with distant metastasis.

**Material and methods:** In order to evaluate the relationship between clinical data and c-erb-B2 and p53 expression, paraffin embedded carcinomas of the oral cavity and oropharynx were prospectively studied by immunohistochemistry. When available, normal mucosa adjacent to carcinoma was also studied.

**Results:** There were 24 patients with oral cavity tumours and 7 with oropharyngeal tumours. c-erb-B2 expression was detected in 12/31 tumors: oral cavity 10/24 (42%) and oropharynx 2/7 (29%). p53 expression was detected in 20/31 tumors: oral cavity 17/24 (71%) and oropharynx 3/7 (43%).

In adjacent mucosa, c-erb-B2 was detected in 13/19 cases: oral cavity 9/14 (64%) and oropharynx 4/5 (80%). p53 expression in normal mucosa was detected in 7/18 cases: oral cavity 6/12 (50%) and oropharynx 1/6 (17%). In 8 cases c-erb-B2 was expressed in adjacent mucosa but not in corresponding tumor.

**Conclusion:** The absence or decreased expression of c-erb-B2 in HNSCC compared to adjacent mucosa could be associated with malignant progression. An inverse relationship between c-erb-B2 and EGFR (also called c-erb-B1) has already been shown in other tumor types and deserve further investigations in HNSCC.

### 1.28

### Absence of c-erb-B2 expression and risk of distant metastasis in pharyngeal tumors

F. Janot, M. Guerry, L. Vabre, M. Talbot, A-M. Le Ridant, G. Mamelle, C. Hill, J. Bosq, B. Luboinski, Inst. Gustave-Roussy, Villejuif, France

**Introduction:** Between 1880 and 1985, 914 patients with head and neck squamous cell carcinoma (HNSCC) underwent lymph node dissection in our institute. The prognostic role of clinical factors has already been reported. We first compared 31 patients with oropharyngeal cancer who developed distant metastases with 31 patients without metastasis, matched on tumor site, nodal site and size, year of diagnosis. Histological differentiation, keratinization, vascular emboli, immunohistochemical expression for bcl2, c-erb-B2, Rb and p53 were determined for the 31 cases and their matched controls. The factors with the lowest p value (p53, c-erb-B2) were further investigated in a larger series.

**Methods:** We added 32 patients with hypopharyngeal cancer who developed distant metastases and 32 matched patients. An exact conditional logistic regression model was used to study the prognostic value of p53 and c-erb-B2 on the risk of distant metastasis.

**Results:** Considering the 63 pairs of patients, the p-value for p53 and c-erb-B2 were 0.18 and 0.047, respectively. Absence of c-erb-B2 expression was associated with an increased risk of distant metastasis.

**Conlculsions:** This study shows that p53 expression is not a strong prognostic factor in HNSCC. The absence of c-erb-B2 expression could be linked with HNSCC progression, as already suggested by Kilpi et al (British J. Dermatol, 133:847-852, 1995).

### 1.30

### tp53 DNA contact mutations are associated with metastasis in head and neck squamous cell carcinoma

R. Erber[1], N. Homann[2], C. Conradt[3], M. Finckh[4], A. Dietz[5], H. Weidauer[5], F.X. Bosch[1], [1,3,5]University of Heidelberg, [4]Clinic Buch, Berlin, Germany, [2]Research Unit of Alcohol Diseases, Helsinki, Finland

**Introduction:** Recent studies have suggested that different mutation types within the core domain of the tumor suppressor protein p53, i.e. DNA contact mutations and conformational mutations, confer different biochemical and biological properties. Here we have analyzed in head and neck squamous cell carcinomas (HNSCC), whether these p53 mutations types have a differential clinical impact.

**Subjects and Methods:** 86 tumors of different anatomical sites and of different clinical stages were subjectet to PCR-based direct sequencing of exons 5-8 of the p53 gene. LOH analysis using microsatellite markers was used to evaluate the allelic status of the p53 gene.

**Results:** 36 missense mutations were identified by PCR-based sequencing of exons 5 - 8. 13 of these (36%) occurred in regions critical for direct DNA contact. Microsatellite marker analysis revealed a selective association between these mutations and the loss of wild-type alleles (100% LOH vs. 50% LOH in the other groups of mutations; p=0.0034). The corresponding tumors had metastasized to a larger extent at the time of diagnosois (92% N+ contact mutated vs. 55% N+ in the other groups of mutations, p=0.0016, ($\chi^2$-test). Both, their disease free survival and overall survival times were significantely shorter as compared to patients with other mutations or patients apparently without mutations.

**Discussion:** These data indicate that in HNSCC, p53 mutations affecting sequenqe specific DNA binding confer a strong selection pressure to eliminate wild-type alleles, and that they result in an accelerated tumor progression, development of metastasis and reduced therapeutic responsiveness.

### 1.31

### p53 antibodies as a prognostic indicator in sera of head and neck squamous cell carcinoma patients

J.R. Kwak[1], T.E. Carey[1], T. Soussi[2], C.R. Bradford[1], J.A Werner[3], [1]University of Michigan, USA; [2]Inst. Curie, Paris, France; [3]University of Kiel, Germany

The role of p53 in cell growth regulation and its frequent overexpression in tumors has lead to studies investigating its involvement in cancer. Mutation of the p53 gene occurs in approximately 45% of human cancers. Mutant p53 may be antigenic since p53 specific antibodies are present in some patients' sera. Furthermore, p53 antibodies may have prognostic significance. The goals of this study are to determine: the frequency of p53 antibodies in a cohort of squamous cell carcinoma (SCC) patients; interlaboratory variability of assays; if antibody titer is predictive for survival; and if there is a correlation with mutations. 719 serial serum samples from 123 SCC patients and 4 control subjects dating from 1978 to 1987 were selected. An ELISA assay using 96 well plates coated with recombinant human p53 protein or control extract from untransfected S9 insect cells was used to determine the p53 specific antibody titer. Collaborating laboratories will test the same samples with other tests. Preliminary results: 27.2% of the samples (190 of 698) and 59.4% of the SCC patients (73 of 123) have p53 antibodies. Correlations between clinical histories and antibody titer fluctuations are being elucidated to assess p53 antibody levels as a prognostic indicator in squamous cell carcinoma.

### 1.32

### Relationship between the cellular DNA content, p53 protein overexpression and metastatic potential of head and neck cancer

L. Tamás[1], H. Kraxner[1], B. Járay[2], O. Ribári[1], G. Répássy[1], Z. Szentirmay[3], [1] Dept. of ORL, [2] 2nd Inst. of Pathology, University of Budapest, [3] National Inst. of Oncology, Budapest, Hungary

**Background/ Aim**: The aim of our study is to examine the relationship between the parameters of cellular DNA content (DNA index, S phase value, polyploid fraction), p53 protein overexpression, histological grading, clinical parameters (age, sex, tumor localization, TNM stage, kind of treatment) and metastatic potential of head and neck cancer.

**Materials and methods:** Patients: 44 patients (7 women, 37 men, average age 59,29) with head and neck carcinomas (38 laryngeal, 4 pharyngeal, 2 oral carcinomas) were treated by operation or irradiation. Pathological procedure: HE staining and p53 immunohistochemistry was performed on 6 μ sections in the tissue samples. The immunohistochemical staining was carried out using monoclonal anti-human p53 antibody (Do 7). Measurement of cellular DNA content: Nuclear suspensions of tissue samples were isolated and stained with Feulgen's reaction. Cellular DNA content was determined by a digital picture analyzing system (DNACE). From each slide about 600 tumor cell-nuclei and up to 100 leukocyte-nuclei (as diploid reference control) were randomly selected. On the basis of histogram measured, the DNA index values and cell cycle parameters (i.e. G1-S-G2 ratio and polyploid fraction) per subfraction were calculated.

**Results:** There is no significant relationship between nor clinical either histological parameters (grading, p53, DNA content) and the evidence of later lymph node metastasis. Correlation was found between p53-positivity and the time of developing lymph node metastasis. (P=0.06). The aneuploid tumors proved to give metastasis more frequently than the euploid ones, but there was no significance. The low (< 15%) or high (> 15%) S-phase fraction (SPF%) value correlates with the metastasis-free time (P=0.05). We found correlation between the low (< 2%) or high (> 2%) value of the polyploid fraction and the time of developing lymph node metastasis (P=0.038).

**Conclusion:** There is a strong correlation between the mutant p53 protein overexpression, the high SPF value, the high polyploid fraction value, and the metastatic potential of head and neck cancer.

### 1.33

**There is no difference in the distribution of flow cytometric (FCM) DNA-indices between node-positive and node-negative squamous cell carcinomas of the head and neck**

J. Wennerberg[1], B. Baldetorp[2], [1]Dept. of ORL/Head and Neck Surgery, [2]Dept. of Oncology, University of Lund, Sweden

Squamous cell carcinomas of the head and neck (HNSCC) evolve from diploid epithelial cells with a normal chromosomal constitution, still 2/3 of HNSCC are non-diploid in FCM analysis and the cells often exhibit a very complex karyotype. At diagnosis one third to one half of HNSCC have clinically positive neck nodes. Presence of neck nodes at diagnosis is a very strong prognostic factor for poor prognosis. The development of a metastatic phenotype requires tumour progression and the acquisition of new properties beyond those necessary for local invasive growth. The question is whether the progression to a metastatic phenotype is reflected in the distribution of FCM DNA indices (DI) in node-negative and node-positive HNSCC.

**Materials & Methods:** Tumour samples from 200 patients with HNSCC were FCM analysed regarding nuclear DNA content using a multistep preparation procedure including RNAse and pepsin treatment, and staining with propidium iodide. Tumors with one stemline were classified as diploid (DI = 1.0) and tumors with two or more stemlines as non-diploid.

**Results:** Close to 2/3 (126/200) were node-negative. T-stage 1-2 were more common in node-negative than in node-positive tumours (52% vs. 35%). The fraction of diploid tumours were higher in the node-negative group (44% vs. 15%), but there was no difference in the frequency distribution of the DNA-indices of the non-diploid tumours in-between the node-negative and node-positive group of tumours.

**Conclusion:** Non-diploidy is thought to be acquired through a multistep process, either by initial loss of chromosomes followed by polyploidisation or by tetraploidisation followed by loss of chromosomal material. Though node-positive tumours are more likely to have a high T stage and be non-diploid than node-negative tumours there is however no differences in-between the groups in the frequency distribution of non-diploid tumours, clustering around a modal value of 1.75. The metastatic phenotype thus does not seem to manifest itself by a change in the amount of nuclear DNA.

### 1.34

**Estimation of DNA adducts level in larynx tumour in relation to cancer staging**

K. Szyfter, Z. Szmeja, W. Szyfter, J. Banaszewski, M. Pabiszczak, Inst. of Human Genetics, Polish Academy of Sciences, Dept. of ORL, University of Poznan, Poland

Tobacco smoke, the main causative agent in larynx cancer, contains several carcinogenic compounds capable to form DNA adducts.

Aromatic DNA adducts formed by polycyclic aromatic hydrocarbons (PAH) and N7-alkylated guanosine derivatives generated by N-nitrosoamines were analysed by HPLC (PAH:DNA) and TLC variants of the $^{32}P$-poslabelling assay. Biological material obtained after partial or total laryngectomy was divided into tumour proper and biopsies recognised as non-malignant. 42 larynx subject biopsies were analysed for N7-alkylated dGMPs and 80 subjects for aromatic DNA adducts.

Large inter-individual variability of DNA adduct content was found in the studied tissues. Nevertheless, it was established that cancer progression is a factor modulating DNA adduct level. Further, it was found that metastasis to adjacent lymph nodes was accompanied by a significant decrease of a level of both types of the studied DNA adducts in tumour cells. The same time the levels of DNA adducts remain almost unchanged in non-tumour cells. There were too few cases of large distance metastasis to study its relationship with DNA adduct level.

The results seem to indicate that tobacco smoke-derived DNA adducts play a significant role at initiation of carcinogenesis but their role at metastasis (albeit not clearly understood) cannot be underestimated.

### 1.35

**Elevated levels of basic fibroblast growth factor in serum and urine of patients with primary head and neck cancer**

S. Tauber, A. Leunig, R. Spaett, M. Leunig[1], G. Grevers, Dept. of ORL, LM University of Munich, Germany; [1]Dept. of Orthopaedic Surgery, University of Bern, Switzerland

**Introduction:** Basic fibroblast growth factor (bFGF) has been suggested to promote angiogenesis, contributing to the growth and metastasis of tumors. Elevated bFGF levels in serum and urine of patients with various types of cancer have been reported. Since no information exists about bFGF levels in patients with primary head and neck cancer, we determined those in the present study.

**Methods:** By use of an immunoassay, bFGF was quantified in the serum and urine of 89 tumor patients (head and neck cancer) and 45 patients with diseases unrelated to cancer. The assay was performed after centrifugation and storage at -80°C.

**Results:** bFGF was detectable in serum and urine of all patients. Cancer patients revealed markedly increased serum bFGF levels ($p<0.014$), advanced tumor size ($T_{3/4}$ vs. $T_{1/2}$) showed significantly increased bFGF levels in both serum and urine ($p<0.037$). There was no association of bFGF concentrations with degree of histologic differentiation (G1-G3).

**Conclusion:** This study demonstrates that bFGF levels are elevated in serum and urine of patients with primary head and neck cancer. These findings suggest an involvement of bFGF in the formation of solid tumors. The quantification of bFGF might be a valuable method for the non-invasive monitoring of treatment response in cancer therapy.

### 1.36

**Epidermal growth factor receptor (EGF-R) bound neuraminic acid alters the cell proliferation in head and neck cancer**

W. Bergler, G. Petroianu, F. Riedel, A. Baker-Schreyer, K. Hörmann, Dept. of ORL, University Hospital Mannheim, Germany

**Purpose:** The overexpression of the EGF-R on squamous cell carcinomas of the head and neck has been connected to malignant transformation but its role for the proliferation of the malignant cell and the factors determine the receptor-ligand interaction are still not clearly defined. The external domain of the EGF-R is known to carry glycan structures with neuraminic acid which might be important for the function of the receptor. Aim of our study was to investigate the role of these sialoglycan structures on the EGF-R for the proliferation of head and neck carcinomas.

**Methods:** On two squamous cell carcinoma cell lines we altered the specific glycostructures with neurominidase from vibrio cholerae (desialylation) and with α-2,6-sialyltransferase and CMP-neurominicacid. Resulting effects were monitored by the cell proliferation with BrdU-method (proliferation assay and cell cycle distribution) and by the EGF-R analysis using I-125 EGF for the Scatchard analysis to determine the receptor affinity.

**Results:** The results showed that the cell proliferation and the receptor affinity is dependent on the degree of sialylation (neuraminic acid). Desialylation led to a 35% reduction of the proliferation, the receptor affinity decreased to 30%.

**Conclusion:** The significance of the EGF-R for the cell proliferation seems to depend on the degree of sialylation. A release of enzyms by the tumor cells could auto-control the tumor proliferation.

### 1.37

**The expression of epidermal growth factor receptor in lymph node metastases of head and neck cancer**

A. Baker-Schreyer, F. Riedel, W. Bergler, K. Götte, G. Petroianu, K. Hörmann, Dept. of ORL, University Hospital Mannheim, Germany

**Purpose:** An overexpression of the epidermal growth factor receptor (EGFR) has been found in a wide variety of malignancies including squamous cell carcinomas of the head and neck. The overexpression of EGFR is of increasing interest because of a possible contribution to metastasis. Primary tumors and metastasis because of a possible contribution to metastasis. Primary tumors and metastasis may differ in the expression of EGFR and provide a basis for metastasis.

**Material and methods:** This study examined the expression of the cell-surface EGFR in frozen tissue samples from 30 primary carcinomas of the head and neck and from 30 lymph node metastases. The examination employed the use of an immunfluorescence assay using a mouse monoclonal antibody for localisation of immunoreactive EGFR.

**Results:** We saw a significant higher expression of EGFR in metastases than in primary tumors (p=0.005). Examining the primaries, no correlation was seen between EGFR level and TNM-stage. We found an interesting correlation between EGFR level and histologic grading, immunoreactivity being significantly higher in G3 than in G1-G2 tumors (p=0.001).

**Conclusions:** EGFR system may play an important role in the process of metastasis and elevated EGFR level might characterize more metastatic tumors. The significant correlation between EGFR level and the histologic grading suggests that EGFR expression may identify biologically more aggressive tumors.

### 1.38

**Epidermal Growth Factor Receptor (EGFR) in squamous cell carcinomas of the head and neck: its role in tumour invasion and potential as a target for therapy**

S. Eccles, H. Modjtahedi, G. Box, W. Court, P. O'charoenrat, C. Dean, McElwain Laboratories, Inst. of Cancer Research, Surrey, U.K.

Squamous cell carcinomas frequently over-express EGFR (type 1 receptor tyrosine kinase) and one or more of its major ligands (EGF, TGFα, amphiregulin). In many studies this has been linked with a higher probability of relapse and relative resistance to chemotherapy and radiotherapy. In addition to providing a simple growth advantage, EGFR activation is implicated in critical processes involved in metastasis; ie adhesion to matrix proteins, migration and up-regulation of matrix metalloproteinases, key enzymes in invasion of tumour cells and penetration of capillary sprouts during angiogenesis.

These observations, plus the accessible cell surface location of EGFR, render it an attractive target for therapy. We generated monoclonal antibodies to 5 epitopes on the external domain of human EGFR, and have shown that they inhibit binding of all ligands. These antibodies inhibit the growth of tumour cells which over-express EGFR, and can induce complete regressions of established human tumour xenografts including carcinomas of the head and neck (eg HN5, HN15). In squamous cell carcinomas we observed a unique mechanism of action; viz induction of terminal differentiation. We are currently deriving new cell lines from primary and secondary head and neck tumours for detailed investigations of the interactions between EGFR, MMPs and their inhibitors in invasion and metastasis. A phase 1 clinical trial of antibody ICR62 is underway.

### 1.39

**Biologic characteristics of metastatic spread in squamous cell carcinomas of the oro- and hypopharynx**

H.-J. Welkoborsky[1], R. Jacob[2], H.S. Bernauer[2], W.J. Mann[2], [1]Dept. of ORL, Nordstadt Clinic, Hannover, [2]Dept. of ORL, University of Mainz, Germany

**Background:** It is a clinical observation that lymphnode metastases of squamous cell carcinomas show a different clinical behavior and a different response to chemotherapy and to radiation therapy than the corresponding primary tumors. Nevertheless the molecular and cytogenetical events leading to metastatic spread are mostly still unknown. The purpose of this study was to evaluate the cytogenetic and molecularbiologic events on which the development of metastastes are depending on.

**Patients and Methods:** Operative specimens of fifty patients who underwent surgery for a squamous cell carcinoma of the oro- or hypopharynx were examined. The examinations included quantitative DNA cytometry, immunohistochemical assessment of proliferation markers and of tumor-suppressor-gene products (i.e. p53, nm-23) along with comparative genomic hybridization (CGH) which could be performed in 25 cases. The results of these examinations in primary tumors and corresponding lymphnode metastases were compared.

**Results:** Tumor cells in lymphnode metastases showed statistcally significant increased values for the parameters of quantitative DNA analysis and proliferation scores when compared to the corresponding primary tumors. Cytogenetically tumor cells in lymphnode metastases showed increased numerical and structural chromosomal aberrations (5.6 chromosomes affected in the average versus 7.8 affected chromosomes). In lymphnode metastases deletions of 18q and amplifications of 3p and 9p were most frequent. On the other hand, the results of immunohistochemical expression of the suppressor gene products p53 and nm-23 were only slightly different.

**Conclusion:** The data demonstrate that tumorcell clones in lymphnode metastases show genetically more aberrations and a higher aneuploidy than in the primary tumors. One might suggest, that only the most aggressive cell clones in primary tumors are responsible for the development of metastatic disease. Therefore tumorcells in lymphnode metastases might reflect a higher stage of the tumor progression cascade.

### 1.40

**Telomerase activity in squamous carcinoma of the head and neck may be indicative for metastatic potential**

D. Thurnher, M. Bakroeva, B. Knerer, M. Formanek, J. Kornfehl, Dept. of Otorhinolaryngology, University of Vienna, Austria

Head and neck cancer arises and progresses through specific alterations which lead to an invasive immortal phenotype. The process of immortalization is associated with the activation of the enzyme telomerase which is capable of synthesizing telomeric repeats at the end of chromosomes. This enzyme is expressed in nearly all neoplasms, germline cells and is absent in most normal human somatic cells. Therefore telomerase activity may deliver useful diagnostic and/or prognostic information about clinical tumor behavior. We analyzed telomerase activity in (1) primary lesions of HNSCC and (2) N0/N+- lymphnodes using a Telomera-se-PCR-Kit, a semiquantitative photometric enzyme immunoassay basing on the telomeric repeat amplification protocol (TRAP). Our data of the primary lesions showed that N0 necks revealed significantly ($p<0.05$) lower emission intensities (i.e. telomerase activity) than N+necks. Preliminary experiments showed that N+lymphnodes express higher telomerase activity than the N0-nodes.

Our results indicate that a higher telomerase activity in HNSCC may faciliate metastatic involvement in lymph nodes and the estimation of telomerase activity could be a useful diagnostic tool possibly influencing treatment modalities.

### 1.41

**All-trans retinoic acid regulates the matrix metalloproteinase (MMP)-mediated invasive behavior of head and neck squamous cell carcinoma cells (HNSCC)**

M.P. Schönermark[1,2], T.I. Mitchell[1], C.E. Brinckerhoff[1], T. Lenarz[1], [1]Dept. of Otolaryngology/Head and Neck Surgery, Hannover Medical School, Germany, [2]Dept. of Medicine, Dartmouth Medical School, Hanover, USA

**Introduction:** To study a potential anti-invasive effect of all-trans retinoic acid (atRA), which has received considerable attention as a chemopreventive agent in head and neck cancer, we studied the impact of atRA-treatment on the expression of MMPs in two HNSCC-cell lines (SCC25 and FaDu) and on the invasion of these cells through a reconstituted basement membrane and through purified collagen type I, respectively.

**Methods:** MMP-gene expression was studied by Northern blotting, MMP-production by Western blotting and SDS-Zymography. To study invasion, a recently introduced quantitative assay was used (*Nature Med 1997; 3:1167-71*).

**Results:** While SCC25 express MMP-1, -2, -3, -9, -13, and MT1-MMP constitutively, FaDu express only MMP-1, -3, -9, and -13. Both cell lines migrate readily through Matrigel. However, only FaDu invasion can be blocked completely by atRA-treatment. This block is concomitant with a downregulation of MMP-9 message and protein. SCC25 invasion through Matrigel is only partly blocked by atRA, suggesting an atRA-resistant subpopulation. Invasion of FaDu cells through collagen I is also blocked by atRA, as is MMP-1 and -13 expression. SCC25, although equipped with all relevant enzymes, are not spontaneously invasive through collagen I.

**Conclusions:** The results indicate that the atRA-induced downregulation of MMP-expression can effectively contribute to a reduction of invasive potential of HNSCC.

### 1.42

**Comparison of tumor biological factors in N0 and N+ pharyngeal and laryngeal squamous cell carcinomas**

R. Jacob, H.-J. Welkoborsky, W.J. Mann, Dept. of Otorhinolaryngology, University of Mainz, Germany

In pharyngeal and laryngeal squamous cell carcinomas the prognosis is determined by the presence or absence of lymph node metastasis. In a prospective study we analyzed the tumor biology of 80 patients (DNA-cytometry, immunohistochemical examination of proliferation markers (PCNA, Ki67-MIB1), wild-type p53, bcl-2, nm23, Integrins and CD44 v4-v6, E-Cadherins and morphological tumor front grading). The aim of this study was to identify tumor biological parameters to estimate the likelihood of lymph node metastasis. The results of the tumor biological results of 30 cases with no evidence of lymph node metastasis were compared with 50 cases with histological confirmed lymph node metastasis (N2 or more). The results show that low 2cDI scores, low proliferation scores and high scores for integrin chain ß3 are associated with a significantly reduced incidence of lymph node metastasis. The other parameters revealed no such association. Based on tumor biological examinations it should be possible to predict the incidence of lymph node metastasis for an inidvidual patient.

### 1.43

**Ultrastructural and biochemical features of larynx cancer with lymph node metastases**

W. Golusinski[1], Z. Szmeja[1], W. Biczysko[2], J. Olofsson[3], Depts. of [1]Otolaryngology and [2]Clinical Pathology, University of Poznan, Poland, [3]Dept. of Otolaryngology, University of Bergen, Norway

An analysis of features of cytoplasm ultrastructure and cellular nuclei of laryngeal cancers was carried out on 120 patients in whom simultaneously DNA-ploidy was performed and immunohistochemical staining for the presence of p53 protein and Ki67 nuclear antigen. Comparing the obtained results of the morphological tests and clinical features special attention was paid to the occurrence of metastases to lymph nodes. These metastases occurred in 58 patients with transglottic and supraglottic tumours, size of the tumour T3 and T4, which were marked histologically as of medium or poor degree of differentiation.

According to the authors the most interesting results concern the group of tumours of medium degree of differentiation. In the electron microscope examination a substantial progression of the malignancy of the tumour in its contour part was revealed, where cells had nuclei which occupied a large area of the cell territory enlarged active and numerous nucleoli and narrow cytoplasm. Cell membrane did not generate junctions and intercellular bridges with the concurrent presence of keratotic pearl exponents of the tumour on the surface or in its centre. To such character of the texture corresponded: strong reaction in the nuclei to the presence of p53 and nuclear antigen Ki67. The evaluation of DNA-ploidy revealed the occurrence of the nuclei with diploidal and polyploidal or aneuploidal-polyploidal DNA patterns in the same carcinomas, which together with the obtained histological results emphasises ,a great importance in the defining the infiltration front. This is particularly connected with the occurrence of metastases to lymph nodes. This group of patients comprised 18 people. The remaining metastases appeared in patients with poor degree of maturity of the tumour and aneuploidal character of DNA-ploidy.

### 1.44

**Histomorphological characteristics of non-metastatic lymph nodes in patients with head and neck cancer according to their site in the neck**

T. Klimek, T. Dreyer, C. Popella, H. Glanz, Dept. of Otorhinolaryngology, University of Gießen, Germany

**Introduction:** The prognostic relevance of histomorphologically defined lymph node reaction patterns was demonstrated in head and neck cancer. Till today the lymph node site in the neck has not been taken into account.

**Material and Methods:** A total of 1188 dissected neck lymph nodes of 32 previously untreated patients with carcinoma of the head and neck region were examined histologically by serial sections and classified into 6 different lymph node levels and 6 histomorphological types. The objectivity of the morphological examinations could be evaluated morphometrically by means of an interactive image analysis system (IBAS).

**Results:** A more precise definition of the lymph node reaction types could be established. The results show a statistically significant relationship between the lymph node reaction types and their site in the neck. These findings allow a division of the neck in an upper anterior half with immunologically active lymph nodes and a lower caudal dorsal half, which contains essentially less immunologically active lymph nodes.

**Conclusions:** The worse prognosis of tumors with lymphatic drainage to the lower parajugular group may not only be explained by the fact that these lymph nodes are situated at the margin of the neck specimen but also by our observation that in these regions primarily lymph nodes with lesser immunological activity are localized.

**1.45**

**Nuclear morphology in tongue carcinomas as a biomarker for the prediction of metastases**

E.J.M. Hannen, J.A.W.M. van der Laak, J.J. Manni, M.M.M. Pahlplatz, H.P.M. Freihofer, P.J. Slootweg, R. Koole, P.C.M. de Wilde, Dept. of ORL, Universities of Nijmegen, Maastricht, Utrecht, The Netherlands

Since cancer is a disease of the genome, and neoplastic progression is associated with accumulation of genetic aberrations, nuclear morphology as assessed by automated, computer assisted image analysis could disclose information as to the extent of the genetic damage.

4 Micron sections of 46 metastasized and 34 non-metastasized tongue carcinomas were Feulgen stained. The aim of this study was to disclose differences in morphology (i.e. nuclear size-, shape-, and chromatin pattern features) between these groups, intending to develop a discriminant function that enables the prediction of metastases for the individual patient.

In the metastasized tumours the chromatin was significantly more condensed ($p=0.01$), and exhibited significantly less variation in chromatin condensation ($p<0.001$) than in the group non-metastasized carcinomas.

Logistic regression analysis resulted in a classifier, based on the circularity of the nucleus (CIRC) and the standard deviation of the chromatin condensation (SD COND), to predict the occurrence of lymph node metastases. After cross validation the percentages of correct classifications in the group of metastasized and non metastasized tumours were 72% and 62%, respectively. Nuclear morphology appeared to be more appropiate to predict metastases than biomarkers such as p53, bcl-2, Ki-67, and the cT-stage.